# Association between genetic mutations and the development of autoimmune thyroiditis in patients with chronic hepatitis C treated with interferon alpha

**DOI:** 10.1186/1756-6614-5-10

**Published:** 2012-10-16

**Authors:** Janina Krupińska, Waldemar Urbanowicz, Mariusz Kaczmarczyk, Grzegorz Kulig, Elżbieta Sowińska-Przepiera, Elżbieta Andrysiak-Mamos, Anhelli Syrenicz

**Affiliations:** 1 Department of Endocrinology, Metabolic Diseases and Internal Diseases, Pomeranian Medical University, Szczecin, Poland; 2 Department of Infectious Diseases and Hepatology, Pomeranian Medical University, Szczecin, Poland; 3 Institute of Clinical Biochemistry and Molecular Diagnostics, Pomeranian Medical University, Szczecin, Poland

**Keywords:** Genetic mutations, Hepatitis C virus, Interferon alpha, Thyroiditis

## Abstract

**Background:**

Considerable progress was made by the introduction of interferon to the treatment of chronic hepatitis C virus infection. This treatment, however, is associated with the risk of developing or exacerbating autoimmune diseases, with chronic autoimmune thyroiditis being one of them. The aim of our study was to evaluate the predisposition to autoimmune thyroiditis in patients with chronic hepatitis C virus during IFN-alpha therapy, depending on the presence of polymorphisms in the promoter region of CTLA-4C (−318)T gene and in exon 1 of A49G gene as well as C1858T transition of PTPN22 gene.

**Methods:**

The study was conducted in 149 patients aged between 18 and 70 years (mean of 43.9 years), including 82 men and 67 women. Control group for the assessment of the distribution of analyzed polymorphism of genotypes consisted of 200 neonates, from whom umbilical blood was drawn for the tests. The patients were divided into three groups: group 1 consisted of 114 patients without thyroid impairment before and during IFN-alpha therapy, group 2 contained 9 patients with AT with the onset prior to IFN-alpha treatment, and group 3 comprised 26 patients with AT starting after the beginning of IFN-alpha therapy.

**Results:**

The frequency of C1858Tand C(−318)T genotypes observed in the study group did not differ significantly from control group. A significant difference, however, was found for A49G polymorphism.

**Conclusions:**

No association was demonstrated between the occurrence of autoimmune thyroiditis with the onset during IFN-alpha therapy and the presence of polymorphisms within CTLA-4 C(−318)T gene in the promoter region and A49G in exon 1, as well as C1858T transition of PTPN22 gene.

## Background

The development of autoimmune diseases is determined by the coexistence of autoimmune, environmental, and genetic factors. Genetic predisposition to autoimmune diseases depends on many genetic loci, of which three play a key role: alleles of major histocompatibility complex genes (human leukocyte antigen- HLA), mainly class II HLA-DR3; alleles of a gene coding cytotoxic T lymphocyte associated antigen-4 (CTLA-4), and alleles of PTPN22 gene coding for lymphoid-specific tyrosine phosphatase (LYP) [1].

CTLA-4 gene, located on chromosome 2q33, codes for membrane protein (cytotoxic lymphocyte antigen 4), which is important for the regulatory functions of T lymphocytes. Some variants of this gene predispose to autoimmune thyroiditis, including Graves’ disease, as well as to type 1 diabetes, Addison’s disease; also, such non-endocrine conditions as rheumatoid arthritis or coeliac disease [2-7]. In order for the autoimmune response to take place, thyroid autoantigens must be presented to immunocompetent cells; class II HLA antigens participate in this process. Many proteins are involved in the activation of T cell dependent on antigen presentation, including CTLA-4, which inhibits this activation. Mutations of CTLA-4 occur spontaneously as a result of DNA copying error during replication or they may be induced by exposure to DNA-damaging chemical factors or viruses incorporated into the genome DNA during the life cycle. The mutation most likely to occur is the erroneous incorporation of a purine or pyrimidine (i.e. A↔G or T↔C substitution) [8]. Three CTLA-4-related polymorphisms have been described to date: substitution of adenine with guanine in exon 1 position 49 codon 17 (CTLA-4 A49G), substitution of thymine with cytosine at position 318 of CTLA-4(T318C) gene promoter, and elongation of AT sequence of microsatellite marker in 3’ untranslated region (3'UTR(AT)n [6,7,9]. The majority of studies focus on the association between exon 1 CTLA-4 gene polymorphism (A49G) and the risk of developing diseases with autoimmune background [10,11].

The inhibition of T cell activation by CTLA-4 is governed by two mechanisms. In the first, activation is inhibited by the signal that constitutes a response to the activation of lymphocyte T receptor. It occurs at the early stage of immune response, when the expression of CTLA-4 and B7 receptor is restricted. Consequently, the inhibition of T cell proliferation and IL-2 secretion also take place. The second mechanism comprises surface competition between CTLA-4 and CD28 for B7 ligand on the antigen presenting cell. This mechanism depends on the expression of CTLA-4 on the surface of T lymphocyte and is known to join immune response at a later stage, when B7 and CTLA-4 expression increases. The consequence of B7 and CTLA-4 binding is the termination of response through restriction of signal from CD28, anergy of activated T lymphocytes, and their subsequent apoptosis. The impairment of CTLA-4 expression and/or function results in abnormal regulation of autoimmune response [12-14].

In 2004, Bottini et al. published the results of a study demonstrating that substitution of cytosine with thymidine at position 1858 of PTPN22 gene is associated with type 1 diabetes in patients from North America and Sardinia [15]. PTPN22 gene is located on chromosome 1p13.3-p13.1 [16]. A 1858T>C polymorphism leads to the substitution of arginine (R) with tryptophan (W) at position 620 (R620W), which makes it impossible for the PTPN22 gene-coded phosphatase to properly bind with Csk signaling molecules [16-18]. Under normal conditions, LYP-Csk complex causes the gradual inhibition of activation signal for T lymphocytes. Apart from polymorphisms within the major histocompatibility complex (MHC) and CTLA-4, a 1858 T>C polymorphism of PTPN22 gene is currently a recognized risk factor for autoimmune diseases [17-20].

The aim of this study was to assess the predisposition to autoimmune thyroiditis in patients with chronic hepatitis C virus treated with IFN-α, depending on the presence of CTLA-4 C(−318)T polymorphism in gene promoter region and exon 1 CTLA-4 gene A49G polymorphism, as well as C1858T transition of PTPN22 gene.

## Methods

### Characteristics of study subjects

The study was approved by the Ethics Committee at Pomeranian Medical University in Szczecin (decision no. KB-0080/88/09).

The study group consisted of 149 patients aged between 18 and 70 years (mean of 43.9 years), including 82 men and 67 women. All subjects were HCV positive and remained under the care of Hepatology Outpatient Clinic at Regional Polyclinical State Hospital in Szczecin between 2003 and 2007. To assess genetic predisposition to autoimmune thyroiditis during IFN-α therapy, C(−318)T polymorphism in gene promoter region and exon 1 CTLA-4 gene A49G polymorphism, as well as C1858T transition of PTPN22 gene were analyzed. Molecular investigations were conducted at the Institute of Clinical Biochemistry and Molecular Diagnostics at Pomeranian Medical University in Szczecin.

The patients were divided into three groups: 1) Group 1 – 114 subjects without impairment of thyroid function before and during IFN-α therapy, 2) Group 2 – 9 subjects with the onset of autoimmune thyroiditis before IFN-α therapy, and 3) Group 3 – 26 patients with the onset of autoimmune thyroiditis after IFN-α therapy.

Detailed clinical characteristics of study subjects can be found in our earlier publication [21].

### DNA isolation

To detect CTLA-4 polymorphisms and PTPN22 transition, 5 ml of venous blood was drawn into Vacutainer test tubes containing 0.1 ml 5% EDTA. The study material consisted of DNA isolated from peripheral blood leukocytes with the use of QIAMP® DNA Mini Kit (Qiagen).

The distribution of analyzed polymorphisms was also assessed in umbilical blood obtained from 200 neonates, who constituted the control group.

### Sequence of starters

Selected polymorphisms of genes: CTLA-4 [rs 231775 A/G (A49G), rs5742909 C/T(C(−318)T)], and PTPN22 [rs2476601 C/T (C1858T)] were identified with PCR-RFLP (polymerase chain reaction-restriction fragment length polymorphism) with the use of specific starter pairs (Table 1).

**Table 1:** Sequence of starters

**Gene**	**Polymorphism**	**Sequence of starters**
*CTLA4*	rs231775	5’-GCTCTACTTCTTGAAGACCT-3’
		5’-AGTCTCACTCACCTTTGCAG-3’
	rs5742909	5’-GGATGCCCAGAAGATTGA -3’
		5’-AAGGAAGCCGTGGGTTTA -3’
*PTPN22*	rs2476601	5’-ACCGCGCCCAGCCCTACTTTTG-3’
		5’-AGCCACCATGCCCATCCCACACT-3’

### Amplification of CTLA-4 and PTPN22 sequences

Optimal PCR conditions for individual starter pairs were established in preliminary experiments (Table 2).

**Table 2:** Amplification conditions

**Gene**	**Polymorphism**	**T**_**annealing**_**[°C]**	**No. of cycles**	**Amplicon [bp]**
*CTLA4*	rs231775	58	37	162
	rs5742909	54	36	215
*PTPN22*	rs2476601	60	37	392

All amplifications were performed in Mastercycler Gradient thermal cycler (Eppendorf).

Amplifications of CTLA4 sequences were performed in 20 μl of reaction mixture containing the following ingredients: 40 ng of genome DNA, PCR buffer [10 mM of Tris–HCl, 50 mM of KCl, and 0.08% of Nonidet P40] (MBI Fermentas), dNTP [200 μM](MBI Fermentas), MgCl2 [1,5 mM](MBI Fermentas), upstream and downstream primer, 4 pmol each (synt. TIB MOLBIOL, Poznań), and 0.5 U of Taq polymerase (MBI Fermentas).

Amplifications of PTPN22 gene sequences were performed in 20 μl of reaction mixture containing: 40 ng of genome DNA, 1 x PCR buffer (QIAGEN), 1 x Q-Solution(QIAGEN), dNTP [200 μM] (MBI Fermentas), upstream and downstream primer, 4 pmol each (synt. TIB MOLBIOL, Poznań), and 1 U of Hot Star Taq polymerase (QIAGEN).

The following temperature-time profile was used in PCR: 1) phase I: preliminary denaturation 94°C – 5 min, 2) phase II (36–37 cycles; Table 2): denaturation: 94°C – 20 sec, annealing: 52-60°C (Table 2) – 40 sec, elongation: 72°C, 3) phase III: final elongation 72°C – 8 min.

Amplification products were subject to restriction analysis with appropriate enzymes (Table 3).

**Table 3:** Conditions of restriction analysis

**Gene**	**Polymorphism**	**Restriction enzyme**	**Restriction fragments [bp]**
*CTLA4*	rs 231775 A/G	Sat I [10 U, 37°C, 12 h]	allele A: 99 + 63
			allele G: 74 + 25 + 63
	rs5742909	Mse I [5 U, 65°C , 12 h]	allele C: 215
			allele T: 124 + 91
*PTPN22*	rs2476601 C/T	Rsa I [5 U, 37°C, 12 h]	allele C: 228 + 74 + 46 + 44
			allele T: 272 + 74 + 46

**Figure 1:**
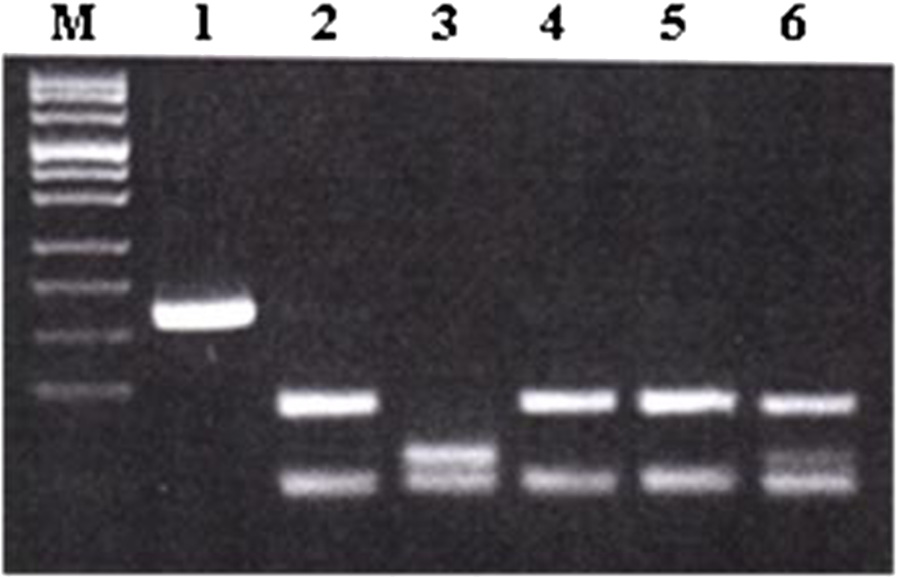
**Identification of RS 231775 polymorphism of CTLA4 gene.** Lanes: M – DNA length marker (Puc Mix Marker 8, MBI Fermentas), 1 – amplicon (162 bp) not subject to restriction, 2, 4, 5 – AA homozygotes, 3 – GG homozygote, 6 – AG heterozygote.

**Figure 2:**
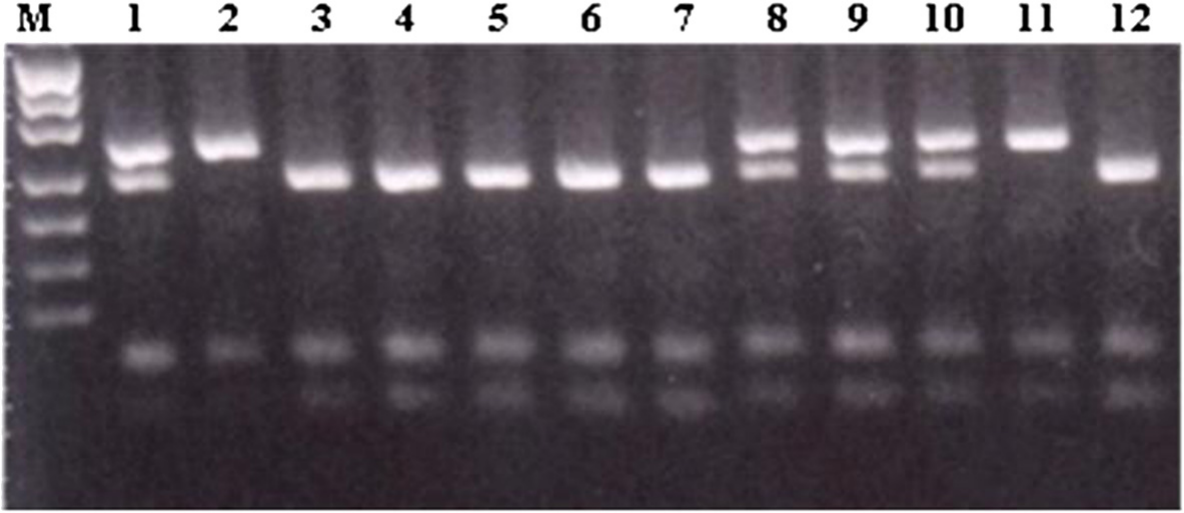
**Identification of rs2476601 polymorphism of PTPN22 gene.** Lanes: M – DNA length marker (Puc Mix Marker 8, MBI Fermentas), 1, 8, 9, 10 – CT heterozygotes, 2, 11 – TT homozygotes, 3, 4, 5, 6, 7, 12 – CC homozygotes.

**Figure 3:**
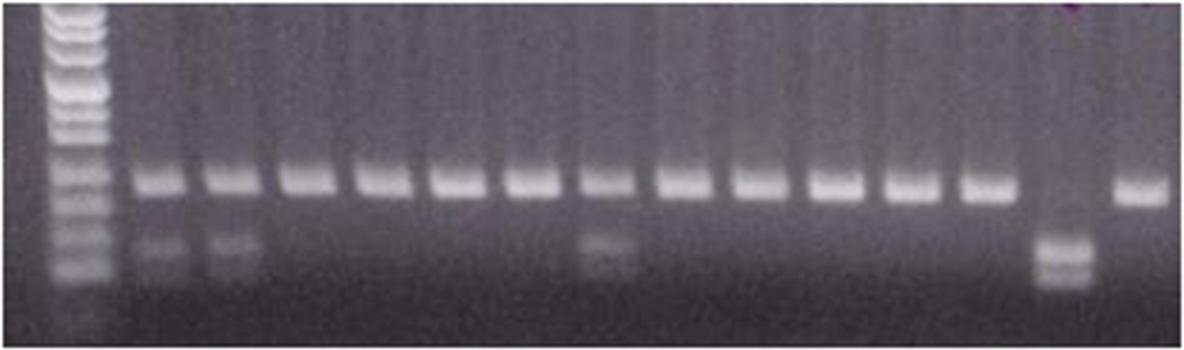
**Identification of rs5742909 polymorphism of CTLA4 gene.** Lanes: M – DNA length marker (pUC Mix Marker 8, MBI Fermentas), 1, 2, 7 – CT heterozygotes, 3, 4, 5, 6, 8, 9, 10, 11, 12, 14 – CC homozygotes, 13 – TT homozygote.

PCR products and restriction fragments were separated by electrophoresis in agarose gel of appropriate concentration and stained with ethidium bromide. The separation was conducted in 1 x TBE buffer (0.089 M of Tris, 0.089 M of boric acid, 2 mM of EDTA), at the temperature of 20°C and voltage of 80V. The length of restriction fragments was estimated on the basis of length marker DNA-pUC Mix Marker 8 (MBI Fermentas). The final stage consisted of photographing the obtained results with Polaroid camera (DS-34 Direct Screen Camera) under UV light (Transiluminator 4000, Stratagene). The photos were scanned and saved as jpeg files (Figures 1, 2, 3).

### Statistical analysis

The conformity of genotype frequency with Hardy-Weinberg law was evaluated with an exact test using R “genetic” module (version 2.9.0; http://cran.r-project.org).

The analysis of polymorphisms was performed with χ2 test and logistic regression model for the calculation of odds ratio (OR) with 95% CI (confidence interval). Allele frequency was calculated on the basis of genotype frequency.

The compliance of genotype frequency with Hardy-Weinberg distribution was analyzed separately for the study group and in the control group, as well as for both of these groups together.

## Results

### Distribution of C1858T, A49G and C(−318)T genotypes in study and control groups

With the exception of A49G polymorphism, genotype frequency did not differ significantly from the theoretical distribution. In the case of A49G polymorphism, the deviations from Hardy-Weinberg equilibrium were found as following for each of the three groups analyzed groups: study group - p<0.0001, control group – p<0.05, study and control groups together - p<0.00001.

The distribution of genotypes of C1858T, A49G, and C(−318)T polymorphisms in the study group and in control group are presented in Table 4. The frequency of C1858T and C(−318)T genotypes in the study group did not differ significantly from that in the control group. A significant difference, however, was observed for A49G polymorphism; moreover, no GG homozygote was detected in the study group, while there were 14 GG homozygotes in the control group, being equal to 7% of all genotypes in this group.

**Table 4:** Distribution of C1858T, A49G, and C(−318)T genotypes in the study group and control group

**SNP**	**Genotypes**	**Study group (n=101)**	**Control group (n=200)**
C1858T	CC	74 (73.2%)	147 (73.5%)
	CT	25 (24.8%)	50 (25.0%)
	TT	2 (2.0%)	3 (1.5%)
	p=0.95347		
A49G	AA	43 (42.6%)	81 (40.5%)
	AG	58 (57.4%)	105 (52.5%)
	GG	0 (0.0%)	14 (7.0%)
	p=0.02423		
C(−318)T	CC	79 (78.2%)	151 (75.5%)
	CT	20 (19.8%)	48 (24.0%)
	TT	2 (1.98%)	1 (0.5%)
	p=0.35636		

### Predisposition to chronic viral hepatitis

The results of analysis in relations to different inheritance modes for minor alleles (dominant and recessive) of the mutated gene and to patient’s gender are presented in Table 5. Dominant mode was defined as the presence of at least one minor allele in comparison to the absence of this allele, resulting in the following three sets of comparisons: TT+CT vs. CC (minor allele T), GG+AG vs. AA (minor allele G) and TT+CT vs. CC (minor allele T), for C1858T, A49G and C(−318)T polymorphisms, respectively. In the case of the recessive mode, homozygotes for minor alleles were compared with the carriers of major alleles, producing the following sets of comparisons: TT vs. CT+CC, GG vs. AG+AA and TT vs. CT+CC for C1858T, A49G, and C(−318)T, respectively.

**Table 5:** Predisposition to chronic viral hepatitis

**SNP**	**Variable**	**OR (95% CI)**	p*
C1858T	TT+CT vs. CC	0.96 (0.56-1.67)	0.895
	TT vs. CT+CC	1.34 (0.22-8.26)	0.749
A49G	GG+AG vs. AA	0.94 (0.57-1.53)	0.799
	GG vs. AG+AA	**---**	**---**
C(−318)T	TT+CT vs. CC	0.87 (0.49-1.54)	0.630
	TT vs. CT+CC	3.82 (0.34-43.3)	0.278

*gender-adjusted p values.

Following gender adjustments, C1858T and C(−318)T polymorphisms, irrespective of the analyzed inheritance mode and A49G polymorphism in the dominant mode (GG+AG vs. AA), did not demonstrate any relationship with the predisposition to chronic viral hepatitis. Odds ratio for A49G in the recessive mode (GG vs. AG+AA) could not be obtained due to the absence of GG homozygotes in the study group, but the observed difference in the frequency of GG homozygotes (0% of patients treated with interferon and 7% of the control group) and allele A carriers (100% of patients treated with interferon and 96% of the control group) was significant (p=0.00334, two-tailed Fisher’s test).

### Distribution of genotypes in patients with autoimmune thyroiditis and in patients without thyroid impairment

Table 6 presents the number and frequency of genotypes of C1858T, A49G, and C(−318)T polymorphisms in patients with autoimmune thyroiditis (AT, n=18) and in patients without impairment of thyroid function during treatment (n=62). The comparison of genotype frequencies in these two groups did not show any significant differences with regards to any of analyzed polymorphisms. It is worth noticing that there were no GG homozygotes of A49G polymorphism, both in AT group and in patients without thyroid impairment.

**Table 6:** Distribution of genotypes in patients with AT and in patients without thyroid impairment

**SNP**	**Genotypes**	**AT (n=18)**	**No thyroid impairment (n=62)**
C1858T	CC	12 (66.7%)	49 (79.0%)
	CT	6 (33.3%)	12 (19.4%)
	TT	0 (0.0%)	1 (1.6%)
	p=0.41034		
A49G	AA	7 (38.9%)	26 (41.9%)
	AG	11 (61.1%)	36 (58.1%)
	GG	0 (0.0%)	0 (0.0%)
	p=0.81720		
C(−318)T	CC	14 (77.8%)	47 (75.8%)
	CT	4 (22.2%)	13 (21.0%)
	TT	0 (0.0%)	2 (3.2%)
	p=0.74153		

### Predisposition to AT during IFN-α therapy

Our study attempted to assess the predisposition to AT depending on the possessed genotype in relation to two inheritance modes and the gender of the patient (Table 7). Since AT group lacked homozygotes of mutated TT C1858T, GG A49G, and TT C(−318)T alleles, the assessment of predisposition to AT in the carriers of these genotypes as compared to the carriers of wild-type (major) alleles (recessive mode) proved impossible. Additionally, A49G GG homozygotes were not detected in patients without thyroid impairment. Conversely, the carriers of mutated (minor) alleles (TT+CT C1858T, GG+AG A49G, and TT+CT C(−318)T) did not show a greater predisposition to AT as compared to wild-type homozygotes (dominant mode).

**Table 7:** Predisposition to AT during IFN-α therapy

**SNP**	**Variable**	**OR (95% CI)**	p*
C1858T	TT+CT vs. CC	1.92 (0.57-6.44)	0.282
	TT vs. CT+CC	---	---
A49G	GG+AG vs. AA	1.26 (0.41-3.89)	0.678
	GG vs. AG+AA	---	---
C(−318)T	TT+CT vs. CC	1.02 (0.27-3.83)	0.971
	TT vs. CT+CC	---	---

*gender-adjusted p values.

OR was not obtained in three cases (recessive inheritance): C1858T – absence of TT homozygotes in the study group vs. one (1.6%) TT homozygote in the control group (p=1.0, two-tailed Fisher’s test); A49G – no GG homozygotes in both groups; C(−318)T – no TT homozygotes in the study group as compared to 2 TT homozygotes (3.2%) in the control group (p=1.0, two-tailed Fisher’s test).

## Discussion

At present, genetic studies are being employed with increasing frequency for the assessment of predisposition to many diseases, especially those that are genetically determined (monogenic and polygenic) as well as those that involve the chromosomes. Diagnostic assessments have become markedly easier to carry out as a result of considerable progress in the investigational methods of molecular biology following the discovery of polymerase chain reaction (PCR) in early 1990s, allowing for the replication of selected DNA fragments in billions of copies. Approximately 80 monogenic diseases have a direct influence on body’s endocrine function; although, polygenic factors are also known to play a role [2].

Genetic polymorphism is the consequence of two types of structural changes in gene sequence, i.e. single nucleotide polymorphism (SNP) and major structural changes such as simple sequence length polymorphism (SSLP), i.e. a variable number of tandem repeats-VNTR (147). According to current genetic research, a common genetic background of autoimmune diseases seems to comprise the following components: C1858T polymorphism of a single nucleotide of PTPN22 gene coding for lymphocyte-specific phosphatase and A/G polymorphism at position 49 of CTLA-4 gene, acting as a negative regulator of lymphocyte T activation, The first results in excessive activation of T lymphocytes, while the second modifies post-translation processes in endoplasmic reticulum through the substitution of threonine with alanine in a signal peptide and this, in turn, causes less effective glycosylation and reduced surface expression of CTLA-4 peptide [22-24]. In 2007, Lee et al. conducted a meta-analysis showing the association between C1858T polymorphism of PTPN22 gene and Graves’ (G) disease, type 1 diabetes, rheumatoid arthritis, and lupus erythematosus [16,22]. Another study published in 2007 demonstrated a very strong causal relationship between 49A/G polymorphism of CTLA-4 gene and the development of Hashimoto Thyroiditis (HT) and the presence of anti-thyroid antibodies in Graves’ disease and HT [25]. A solid correlation between autoimmune thyroid diseases and CTLA-4 polymorphism on chromosome 2q33 was proved by Bicek et al. in 2009, namely two A>G polymorphisms of a single nucleotide of CTLA-4 gene at position +49 of exon 1 (49A/G) and +6230 (CT60) in 3’UTR [26]. We analyzed the distribution of genotypes of C1858T, A49G, and C(−318)T polymorphisms in the study subjects and in the control group. A significant difference was found for A49G polymorphism in the study group vs. control group, while the frequency of C1858T and C(−318)T genotypes did not differ significantly in both groups. The aim of our study was to assess the occurrence of C(−318)T polymorphism of CTLA-4 gene within the promoter region. Additionally, we aimed to evaluate A49G polymorphism in exon 1 and C1858T transition of PTPN22 gene in a group of patients with the onset of AT during INF-α therapy for HCV as compared to subjects without any impairment of thyroid function during treatment. The comparison of genotype frequency between these groups conducted in our study showed no significant differences for all polymorphisms. This result differs from that reported by Kula et al. in 2003, who found the strongest association between CTLA-4 polymorphism and the development and clinical picture of HT in a group of 89 Polish subjects [27]. 49A/G polymorphism associated with the activation of T lymphocytes influences the high level of anti-thyroid antibody production in G-B disease and HT [23,28]. Kula et al. [27] did not find any significant differences in mean TPOAb level for CTLA-4 gene in relation to the genotype with demonstrated association with the exacerbation of hypothyroidism and thyroid volume. Slovenian investigators examined 328 Caucasian patients with G-B disease and Postpartum Thyroiditis (PPT), and compared the results with those obtained in a control group of 117 healthy subjects. The distribution of genotypes of both polymorphisms was similar in these two groups. The frequency of GG genotype in G-B disease was 13.8% for 49A/G polymorphism as compared to 5.1% in the control group; the corresponding values for CT60 polymorphism were 40.7% in the study group and 25.6% in the control group. The frequencies of GG genotype for HT and PPT were comparable and were found to equal to 12.9% for 49AG polymorphism and 34.4% for CT60 polymorphism. A comparison of G allele vs. A allele for both polymorphisms within CTLA-4 gene was also performed and showed that the likelihood of developing G-B disease is 1.6 times greater for individuals with G allele in 49A/G or CT60 polymorphism of CTLA-4 gene. It was demonstrated that 49A/G and CT60 polymorphisms of CTLA-4 gene display strong causal relationship with autoimmune thyroid diseases (AITD) in Slovenian population [26]. Additionally, a correlation between the presence of GG genotype and the development of HT and PPT were also showed [11,26]. We evaluated the frequencies of genotypes in patients treated with INF-α and in the control group and, as mentioned before, a significant difference was observed for A49G polymorphism in both groups. Namely, no GG homozygotes were detected in the study group as compared to the control group with 14 GG homozygotes, constituting 7% of all genotypes in this group. Furthermore, A49G GG homozygotes were not found in patients without thyroid impairment. Conversely, in a meta-analysis published by Roycroft et al. in 2009, which examined C1858T polymorphism of PTPN22 gene in the population of Caucasian patients with Addison’s disease living in Britain and in Poland [29], sixty-one out of 502 (12.2%) British patients were the carriers of T allele in PTPN22 C1858T(R620W)SNP. This allele was detected in 67 (7.8%) of 858 healthy subjects from the control group. In the Polish population of 174 patients, 1858T alleles were found in 34 (19.5%) subjects compared with 11.7% in the control group. The authors performed another comparison within this study, namely, they compared the frequency of 1858T allele in control groups originating from different parts of Europe and obtained the following results: Newcastle (England) – 7.8%, Sheffield (England) – 10.5%, and Norway – 10.8%. Therefore, it is our opinion that the correlation between the polymorphism of PTPN22 gene and the occurrence of Addison’s disease in European population cannot be confirmed beyond a reasonable doubt as authors claim [29].

Ever since the relationship between CTLA-4 polymorphisms and G-B disease and type 1 diabetes in southern European countries was demonstrated for the first time, numerous studies have been conducted to link a given polymorphism with a specific disease. So far, many authors have stressed a strong association between G-B disease and the polymorphism of CTLA-4 gene; however, a considerable controversy has arisen as to the site of polymorphism. For example, Bicek et al., state that CT60 polymorphism shows the strongest correlation only with G-B disease and that its association with HT and PPT is weaker [26]. Petrone et al. demonstrated that in Italian population A49G polymorphism was correlated with G-B disease, especially when G allele was present [30]. Ban et al. observed a much greater frequency of G allele SNP CT60 associated with G-B disease in Japanese subjects than in Caucasian population: 72.6% vs. 52.3%, respectively [31]. In Japanese population, the presence of G allele CT60 was more common in patients with G-B disease than in the control group: 84.0% and 72.6%, respectively; just as it was in the whole group of patients with autoimmune thyroid diseases: 80.1% and 72.6%, respectively. A dominant genotype model among AITD patients was GG+GA vs. AA, while a recessive genotype model was found to be GG vs. AG+AA [32]. We tried to analyze the various modes of inheritance of mutated allele, i.e. dominant inheritance (a necessary condition was the presence of at least one mutated allele versus the absence of this allele), producing three sets of comparisons: TT+CT vs. CC (mutated T allele), GG +AG vs. AA (mutated G allele), and TT+CT vs. CC (mutated T allele) for C1858T, A49Gm and C(−318)T polymorphisms, respectively. Recessive inheritance (the presence of both mutated alleles was necessary) resulting in the following genotypes: TT vs. CT+CC, GG vs. AG+AA, and TT vs. CT+CC for C1858T, A49G, and (C-318T) polymorphisms, respectively, was also analyzed. We found that the carriers of mutated alleles (TT+CT C1858T, GG +AG A49G, and TT+CT C(−318t)) did not have a higher predisposition to autoimmune thyroiditis (AT) as compared to homozygotes of dominant mode. We also tried to assess the predisposition to AT depending on the genotype possessed, taking into consideration two different modes of inheritance and patient’s gender. However, since AT group lacked homozygotes of mutated alleles: TT C1858T, GG A49G, and TT C(−318)T, the evaluation of predisposition to AT in the carriers of these genotypes as compared to the carriers of wild-type alleles (recessive mode) was not feasible in our study. Kucharska et al. reports that among 68 HT patients, G allele and homozygotes with rare G/G allele were significantly more frequent. These authors found no significant difference in the occurrence of homozygotes with common A/A allele and heterozygotes [9]. Kinjo et al. demonstrated that in a group of 144 patients with G-B disease, the frequency of AA, AG, and GG genotypes at position 49 of CTLA-4 gene was significantly higher than in the control group [28]. The frequency of GG genotype was significantly greater, while the frequency of AA genotype was significantly lower in patients with G-B disease as compared to the control. In 1997, Witas et al. analyzed the occurrence of polymorphic alleles of CTLA-4 gene in Polish population of 122 children and found C(−318)T polymorphism in 20.5% of analyzed subjects. Their data indicates that the frequency of T allele was 0.107 and estimated occurrence of heterozygotes 19.1% [33]. The study conducted in Germany by Deichmann et al. in a group of 239 patients with G-B disease demonstrated that C(−318)T polymorphism was found in 13.4% of patients, and heterozygotes were detected in 23.2% of cases [17]. The studies of genetic predisposition to autoimmune endocrine diseases, including autoimmune thyroiditis, focus on the search for polymorphisms within candidate genes responsible for autoimmune diseases. In Hashimoto’s autoimmune thyroiditis, apart from known genetic liability related to the presence of histocompatibility antigens class II DR 3, 4 and 5, and the recognized effect of such environmental factors as the excess of iodine or medications (e.g. interferon), the presence of polymorphisms within CTLA-4 gene and PTPN22 gene must also be taken into account. The results of studies conducted to date are inconclusive; therefore, further research is necessary to explain the etiopathogenesis of autoimmune thyroid diseases.

## Conclusions

No association was found between the occurrence of autoimmune thyroiditis during interferon therapy and the presence of polymorphisms within CTLA-4 C(−318)T gene in the promoter region and A49G in exon 1, as well as C1858T transition of PTPN22 gene.

## Competing interests

The authors declare that they have no competing interests.

## Authors’ contributions

JK and AS conceived of the study, participated in its design and coordination, and helped to draft the manuscript. WU, GK, ESP and EAM conceived of the study and helped to draft the manuscript. MK carried out the molecular genetic studies and participated in the design and coordination of the study. All authors read and approved the final manuscript.
